# Comparative Effectiveness of Chemotherapy Alone Versus Radiotherapy-Based Regimens in Locally Advanced Pancreatic Cancer: A Real-World Multicenter Analysis (PAULA-1)

**DOI:** 10.3390/curroncol30060427

**Published:** 2023-06-10

**Authors:** Alessandra Arcelli, Giuseppe Tarantino, Francesco Cellini, Milly Buwenge, Gabriella Macchia, Federica Bertini, Alessandra Guido, Francesco Deodato, Savino Cilla, Valerio Scotti, Maria Elena Rosetto, Igor Djan, Salvatore Parisi, Gian Carlo Mattiucci, Michele Fiore, Pierluigi Bonomo, Liliana Belgioia, Rita Marina Niespolo, Pietro Gabriele, Mariacristina Di Marco, Nicola Simoni, Johnny Ma, Lidia Strigari, Renzo Mazzarotto, Alessio Giuseppe Morganti

**Affiliations:** 1 Radiation Oncology, IRCCS Azienda Ospedaliero-Universitaria di Bologna, 40138 Bologna, Italyjohnny.ma@studio.unibo.it (J.M.);; 2 Dana Farber Cancer Institute, Harvard Medical School, Boston, MA 02215, USA; 3 Radioterapia Oncologica ed Ematologia, Dipartimento Universitario Diagnostica per Immagini, Università Cattolica del Sacro Cuore, 00168 Rome, Italy; 4 Radioterapia Oncologica ed Ematologia, Dipartimento di Diagnostica per Immagini, Fondazione Policlinico Universitario “A. Gemelli” IRCCS, 00168 Rome, Italy; 5 Radiation Oncology, Department of Medical and Surgical Sciences—DIMEC, University of Bologna, 40138 Bologna, Italy; 6 Radiation Oncology Unit, Gemelli Molise Hospital, Università Cattolica del Sacro Cuore, 86100 Campobasso, Italy; 7 Radiation Oncology Department, Centro di Riferimento Oncologico di Aviano (CRO) IRCCS, 33081 Aviano, Italy; 8 Medical Physics Unit, Gemelli Molise Hospital, Università Cattolica del Sacro Cuore, 86100 Campobasso, Italy; 9 San Rossore Private Hospital, 56122 Pisa, Italy; 10 Radiotherapy Unit, Ospedale Belcolle, 01100 Viterbo, Italy; 11 Institute of Oncology Vojvodina, Sremska Kamenica, Medical Faculty, University of Novi Sad, 21204 Novi Sad, Serbia; 12 Radiotherapy Unit, Ospedale Casa Sollievo della Sofferenza, 71013 San Giovanni Rotondo, Italy; 13 Radiation Oncology, Mater Olbia Hospital, 07026 Olbia, Italy; 14 Research Unit of Radiation Oncology, Department of Medicine and Surgery, Università Campus Bio-Medico di Roma, 00128 Rome, Italy; 15 Operative Research Unit of Radiation Oncology, Fondazione Policlinico Campus Bio-Medico di Roma, 00128 Rome, Italy; 16 Radiation Oncology, Azienda Ospedaliero Universitaria Careggi, 50134 Florence, Italy; 17 Department of Radiotherapy, IRCCS Ospedale Policlinico San Martino, 16132 Genova, Italy; 18 Health Science Departmente (DISSAL), University of Genoa, 16132 Genova, Italy; 19 Radiation Oncology, Fondazione IRCCS San Gerardo dei Tintori, 20900 Monza, Italy; 20 Department of Radiotherapy, Fondazione del Piemonte per l’Oncologia (FPO), IRCCS Candiolo, 10060 Candiolo, Italy; 21 Oncology Unit, Department of Medical and Surgical Sciences—DIMEC, University of Bologna, S. Orsola-Malpighi Hospital, 40138 Bologna, Italy; 22 Radiotherapy Unit, Azienda Ospedaliera Universitaria, 43126 Parma, Italy; 23 Department of Medical Physics, IRCCS Azienda Ospedaliero-Universitaria di Bologna, 40138 Bologna, Italy; 24 Radiotherapy Unit, University Hospital, 37126 Verona, Italy

**Keywords:** pancreatic cancer, chemotherapy, stereotactic body radiotherapy, conventionally fractionated radiotherapy, chemoradiation

## Abstract

Different options for locally advanced pancreatic cancer (LAPC) are available based on international guidelines: chemotherapy (CHT), chemoradiation (CRT), and stereotactic body radiotherapy (SBRT). However, the role of radiotherapy is debated in LAPC. We retrospectively compared CHT, CRT, and SBRT ± CHT in a real-world setting in terms of overall survival (OS), local control (LC), and distant metastasis-free survival (DMFS). LAPC patients from a multicentric retrospective database were included (2005–2018). Survival curves were calculated using the Kaplan–Meier method. Multivariable Cox analysis was performed to identify predictors of LC, OS, and DMFS. Of the 419 patients included, 71.1% were treated with CRT, 15.5% with CHT, and 13.4% with SBRT. Multivariable analysis showed higher LC rates for CRT (HR: 0.56, _95%_CI 0.34–0.92, *p* = 0.022) or SBRT (HR: 0.27, _95%_CI 0.13–0.54, *p* < 0.001), compared to CHT. CRT (HR: 0.44, _95%_CI 0.28–0.70, *p* < 0.001) and SBRT (HR: 0.40, _95%_CI 0.22–0.74, *p* = 0.003) were predictors of prolonged OS with respect to CHT. No significant differences were recorded in terms of DMFS. In selected patients, the addition of radiotherapy to CHT is still an option to be considered. In patients referred for radiotherapy, CRT can be replaced by SBRT considering its duration, higher LC rate, and OS rate, which are at least comparable to that of CRT.

## 1. Introduction

The five-year overall survival (OS) rate of pancreatic cancer patients ranges between 7% and 10% [1]. Moreover, pancreatic cancer is estimated to become the second leading cause of cancer-related mortality before 2030 [2]. Locally advanced pancreatic cancer (LAPC) is an unresectable disease that represents 30–40% of newly diagnosed pancreatic cancer, and which has an intermediate prognosis between resectable and metastatic patients [3].

International guidelines suggest several therapeutic options in LAPC, such as chemotherapy (CHT) alone, CHT followed by conventionally fractionated chemoradiation (CRT), and stereotactic body radiotherapy (SBRT), possibly combined with CHT. [4] SBRT is an emerging technique in LAPC, allowing reduced irradiation of healthy organs and a very short treatment duration.

CHT alone and CHT plus CRT were compared in phase III randomized trials with conflicting results [5,6,7], while SBRT was never tested in randomized trials on LAPC. In fact, some phase II trials showed OS and local control (LC) rates almost comparable to CRT or CHT, with a good short-term toxicity profile [8,9,10,11]. Furthermore, a systematic review reported improved 2-year OS after SBRT (26.9%) compared to standard CRT (13.7%) in this setting (*p* = 0.004) [12]. However, more data are needed to definitively assess the potential role of SBRT in LAPC [13]. Moreover, real-world data comparing treatment options available in this setting are lacking.

Based on this background, we retrospectively compared CHT alone, CRT +/− CHT, and SBRT +/− CHT in LAPC patients, with an updated follow-up in terms of OS, LC, and distant metastasis-free survival (DMFS). We performed an observational, multicenter, retrospective study on a large database including only LAPC patients.

## 2. Materials and Methods

### 2.1. Study Design

This was a retrospective observational multicenter study (PAULA-1) that included LAPC patients from 15 institutions in Italy, including academic and non-academic centers. Eight centers had a Surgery Department dedicated to pancreatic surgery with >20 pancreatic resections per year. Patients were treated between January 2005 and March 2018, either with CHT alone, CRT (delivered with conventionally fractionated RT) +/− CHT, and SBRT +/− CHT, with an updated follow-up. The endpoints of the analysis were OS, LC, and DMFS.

### 2.2. Eligibility

In this analysis we included LAPC patients (clinical stage T3–4) not previously treated with radiotherapy or chemotherapy, not previously or subsequently treated with abdominal surgery, and without distant metastases (Figure S1). Patients were assigned to the LAPC category, preferably after discussion within a multidisciplinary board. We admit that the definition of “LAPC” based on vascular involvement can vary between different institutions, according to the volume of surgery departments and to the different expertise of surgeon and radiologists involved in the discussion of clinical cases.

Only patients with cytologic or histologic evidence of LAPC adenocarcinoma were included, while subjects with mucinous cystadenocarcinoma, small-cell carcinoma, or islet cell or papillary cystic neoplasm were excluded. 

The American Joint Committee on Cancer’s eighth edition staging system was used to classify patients. Patients with lymph node metastases were considered to be those with a nodal short axis more than 10 mm at contrast-enhanced CT scan. 

### 2.3. Treatment

Details of SBRT and CRT planning and delivery techniques were previously described [14,15]. Patients treated with CHT, alone or with CRT, received gemcitabine- or fluoropyrimidine-based regimens. CRT and SBRT were combined with CHT in most patients. The choice of therapy was based on the discussion of clinical cases within multidisciplinary teams.

### 2.4. Follow-Up

Regular follow-up examinations were carried out three weeks after the treatment and every 3–4 months thereafter. Patients were followed up with routine blood tests, Ca19-9, and contrast-enhanced CT scan. Local disease progression was defined as a >20% increase in the sum of tumor diameters from the baseline, or as the appearance of new metastatic lesions in regional lymph nodes. The diagnosis of local progression or distant metastases during follow-up was based on periodic contrast-enhanced CT scans, supplemented by 18F-FDG-PET/CT and/or MRI in equivocal cases, with no need for pathological confirmation. 

### 2.5. Statistical Analysis

Descriptive statistics included median and percentages for continuous and categorical variables, respectively. Continuous variables were compared in descriptive analysis with the Kruskal–Wallis test. Patient and treatment characteristics among the three patient cohorts were compared using the Chi-square test. Kaplan–Meier survival curves [16] were calculated from the initiation of CHT (in patients receiving CHT alone) and from the initiation of radiotherapy (in patients treated with CRT or SBRT), and were tested with the log-rank test [17]. In univariate analysis, we tested the prognostic impact of the following parameters: treatment, age, gender, performance status assessed using the Eastern Cooperative Oncology Group (ECOG) score for cancer patients by oncology healthcare professionals, tumor site (pancreatic head, body, tail—based on the CT scan report) and diameter, clinical tumor and nodal stage. Considering the inclusion in this study of only LAPC (cT3-4) patients, the tumor diameter was analyzed using the values of 3 and 4 cm as the cut-off. With the aim to identify predictors of LC, OS, and DMFS, multivariate Cox’s proportional hazards ratios [18] were calculated to estimate the independent effects of all parameters with a statistical significance level < 0.1 at univariate analysis. Proportionality assumption in the Cox model was checked with the scaled Schoenfeld residuals for each covariate, correlating the corresponding set of scaled Schoenfeld residuals with time, to test for independence between residuals and time. A *p*-value < 0.05 was considered statistically significant. Patients lost to follow-up were analyzed up to the time of dropout and then excluded from statistical analysis. Statistical analysis was performed with IBM SPSS (IBM SPSS Statistics for Windows, Inc., Version 28.0; IBM Corp, Armonk, NY, USA) and with MedCalc version 22.001.

### 2.6. Ethical Issues

The institutional review boards of the participating centers approved the study protocol (201/2015/O/OssN). All enrolled patients signed a written informed consent.

## 3. Results

### 3.1. Patients and Treatment Characteristics

Four hundred and nineteen patients were included in this analysis. Thirty-two patients (7.6%) were lost to follow-up. Their characteristics are reported in Table 1. 

Median follow-up was 16.6 months (range: 3.0–92.0). Two hundred and ninety-eight patients were treated with CRT (71.1%), 65 (15.5%) with CHT alone, and 56 (13.4%) with SBRT. Of the patients treated with CRT and SBRT, 71.8% and 73.2% received CHT before or after radiotherapy, respectively. Patients treated with CHT alone had a significantly higher rate of subjects with ECOG performance status 1–2 (compared to ECOG 0; *p* = 0.005), with tumor in the pancreatic tail (*p* = 0.004), with clinical T4 stage (*p* = 0.012) and clinical N1 stage (*p* < 0.001). Across the treatment cohorts, gemcitabine- and fluoropyrimidine-based regimens were used in 56.8% and 35.3% of patients, respectively. Further details on the CHT regimens administered in the analyzed patient cohorts are reported in Paragraph S2. The median radiotherapy total dose was 30.0 Gy (range: 18.0–45.0) in the SBRT cohort, with 48.0 Gy median BED_α/β10_ (range: 28.0–78.7). The median total dose was 50.4 Gy (range: 10.8–66.0) in the CRT cohort, with 59.4 Gy median BED_α/β10_ (range: 12.7–115.1).

### 3.2. Outcomes

#### 3.2.1. Local Control

In univariate analysis, both patients with tumors of the pancreatic body (*p* = 0.020) and subjects treated with SBRT or CRT (*p* < 0.001) showed prolonged LC (Table 2).

**Figure 1:**
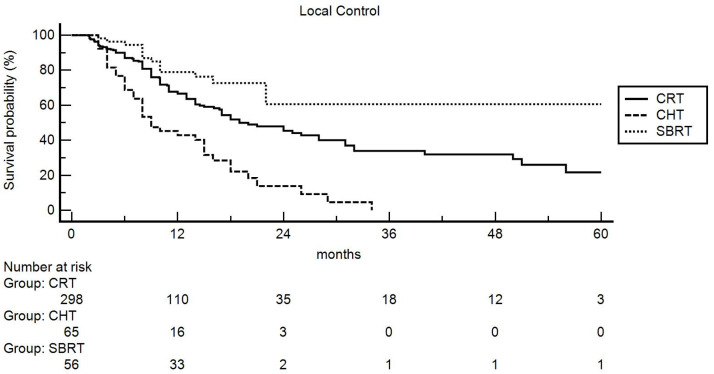
Actuarial local control: comparison between chemoradiation (CRT), chemotherapy (CHT), and stereotactic body radiotherapy (SBRT).

The multivariable analysis (Table 2) comparing the three therapeutic options confirmed the higher LC rates obtained at univariate analysis (Figure 1), compared to patients undergoing CHT alone, in subjects treated with both CRT (HR: 0.56, _95%_CI 0.34–0.92, *p* = 0.022) and SBRT (HR: 0.27, _95%_CI 0.13–0.54, *p* < 0.001).

The impact (statistical significance or trend) of the tumor site and cN stage was not confirmed. Furthermore, excluding patients treated with CHT, subjects undergoing SBRT showed higher LC rates compared to patients treated with CRT (HR: 0.46, _95%_CI 0.25–0.83, *p* = 0.011). Moreover, the higher LC rate in patients with LAPC in the pancreatic body was also confirmed (HR: 0.53, _95%_CI 0.32–0.88, *p* < 0.015).

#### 3.2.2. Distant Metastasis-Free Survival

In univariate analysis, patients with cT4 tumors (*p* < 0.001) showed significantly prolonged DMFS rates (Table 3). However, at multivariable analysis, patients with cT4 LAPC showed only a statistical trend for prolonged DMFS, while no differences were recorded between the three treatment cohorts. Moreover, the correlations between DMFS and gender were not confirmed.

#### 3.2.3. Overall Survival

**Figure 2:**
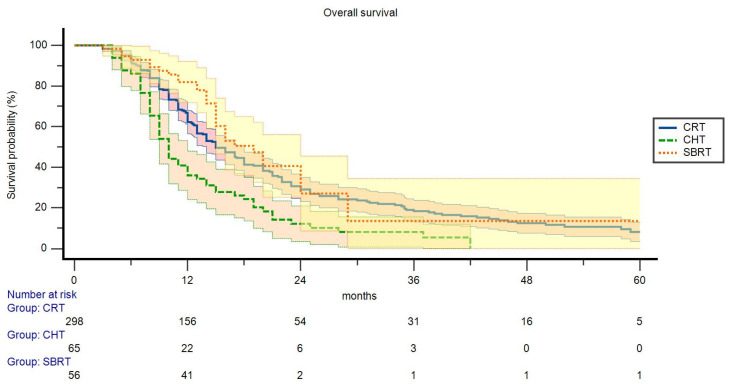
Actuarial overall survival: comparison between chemoradiation (CRT), chemotherapy (CHT), and stereotactic body radiotherapy (SBRT).

In univariate analysis, patients with lower Ca19-9 levels (below the median value of our cohort) and with LAPC in the pancreatic tail showed improved OS (*p* = 0.042 and *p* = 0.025, respectively), while patients with ECOG 2 performance status showed significantly worse OS compared to patients with ECOG 0–1 (*p* = 0.007). Moreover, compared to patients treated with CHT alone, subjects receiving CRT and SBRT showed improved OS (*p* < 0.001) (Table 4, Figure 2).

However, comparing patients treated with CHT alone and patients undergoing SBRT alone or CRT alone (without CHT before and after irradiation), we did not record significant differences in terms of median survival (CHT alone: 10.0 months; SBRT alone: 11.8 months; CRT alone: 14.0 months; *p* = 0.323).

The multivariable analysis comparing the three therapeutic options showed prolonged OS compared to patients undergoing CHT alone, in subjects treated with both CRT (HR: 0.44, _95%_CI 0.28–0.70, *p* < 0.001) and SBRT (HR: 0.40, _95%_CI 0.22–0.74, *p* = 0.003). Moreover, the higher OS rates in patients with LAPC in the pancreatic tail were also confirmed (HR: 0.30, _95%_CI 0.11–0.82, *p* = 0.019). The correlation of OS with ECOG, tumor diameter, Ca 19.9 levels, cT, and cN stage, were not confirmed. Furthermore, excluding patients treated with CHT alone, subjects undergoing SBRT did not show significant differences compared to patients undergoing CRT.

## 4. Discussion

### 4.1. Summary and Contributions

We performed a real-world analysis of clinical outcomes in patients with LAPC treated with three different therapeutic options updating their follow-up data. The analysis showed results comparable to those reported in randomized trials. In patients undergoing CHT alone, the median OS was 10 months, which is within the range of the results reported in three randomized trials (9.2–16.5 months) [5,6,7]. Likewise, the median OS was 15 months in patients treated with CRT, similar to the same randomized trials (range: 8.6–15.2 months) [5,6,7,19]. Moreover, the median OS was 19 months in patients treated with SBRT, similar to the results reported in a meta-analysis (median OS: 17 months) [20].

Our analysis also showed that patients undergoing radiotherapy (CRT or SBRT) +/− CHT had better prognostic characteristics compared to patients undergoing CHT alone. This difference could explain the better results in terms of OS recorded in patients undergoing radiotherapy +/− CHT compared to those undergoing CHT alone. Randomized trials evaluating the impact of combining CHT with CRT showed conflicting results, with one trial reporting a detrimental effect of CRT [5], one showing an improvement in progression-free survival but not in OS [6] after CRT, and finally, one recording a significantly improved OS in patients treated with the combined modality treatment [7].

### 4.2. Strenght and Limitations

Even within the limits of a non-randomized study, our analysis confirms the positive results of the latter trial [7], or at least that the addition of CRT to CHT improves LC, does not have a negative effect on OS, and can improve both median and long-term OS in selected patients. Indeed, not only was median OS prolonged in patients treated with CRT and SBRT (15 and 19 months, respectively) compared to CHT alone (10 months), but also the 4-year OS rates were higher in patients treated with CRT and SBRT (12.4% and 13.5%, respectively), compared to CHT alone (0.0%). For these reasons, further studies are needed to clarify the impact of the combination of radiotherapy with CHT, and above all to define the patient population who can obtain a significant advantage from this combined modality treatment, both in terms of outcome and quality of life. However, it should be stressed that the results of our comparisons should be considered with caution, considering the heterogeneity of our patient series. For example, the CHT regimens used in the CHT cohort were different from those administered in the SBRT and CRT cohorts, although this difference did not reach statistical significance.

In terms of comparison between CRT and SBRT, our analysis confirms the results of some studies reporting a higher LC rate after SBRT [15,21]. Conversely, the difference between the median OS recorded after CRT and SBRT (15 vs. 19 months, respectively) was not statistically significant, unlike some studies comparing matched cohorts [22,23], and one meta-analysis [12] showing a significant benefit in patients treated with SBRT. This difference could be explained by the different design of the latter studies compared to our analysis, together with the relatively small sample size of our SBRT cohort.

Furthermore, it should be noted that the radiotherapy dose delivered in the SBRT cohort was quite low (median BED_α/β10Gy_: 48 Gy) compared to some studies showing a clinical benefit in patients undergoing SBRT with a BED _α/β10 Gy_ > 70 Gy [24] or > 100 Gy [25]. It should also be noted that the follow-up of our patients was based only on contrast-enhanced CT scans without a centralized imaging review, and that the limitations of CT scans in the local evaluation of pancreatic cancer after radiotherapy are well known [26]. Another unexpected result of our analysis is the higher metastasis-free survival rate in patients with cT4 compared to cT3 stage. This data suggests that tumors growing more locally without developing hematogenous metastases, and are intrinsically less metastatic for biological reasons. The identification of factors predictive of only local tumor development would be useful in selecting patients to undergo local consolidative therapies, such as radiotherapy, after systemic treatment. Wilson et al. [27] reported that patients with less FDG-avid pancreatic tumors are less likely to metastasize, and may therefore benefit from upfront local treatment intensification. Therefore, future research in this area seems warranted.

Our study has several limitations. The retrospective design of this analysis is inherently related to a risk of selection bias, which is only partially corrected by the multivariable analysis. Considering the prolonged enrollment period (2005-2018), it is possible that some patients, especially in the CHT-only cohort, may have been treated with systemic regimens that are now considered obsolete, i.e. gemcitabine. Moreover, patients undergoing definitive CHT had poorer prognostic characteristics in terms of ECOG, cT, and cN (Table 1). Indeed, a recent metanalysis [28] of 11 studies showed 24.2 months pooled the median OS in 315 LAPC patients treated with FOLFIRINOX, longer than previous data reported with gemcitabine (11–13 months) [5,7]. Moreover, two recent phase II randomized trials comparing modified FOLFIRINOX versus gemcitabine and nab-paclitaxel [29], or versus gemcitabine plus oxaliplatin and 5-FU [30] in LAPC, provided similar clinical outcomes and efficacy. Conversely, SBRT patients were more likely to receive those “modern CHT regimens”, considering the relatively recent introduction of this technique. 

Furthermore, some data were missing; in particular, Ca19-9 levels (33.2%), the cN stage (5.2% of patients), and dose/fractionation (15.5% of patients). It was not possible to analyze the impact of different treatments on resectability, acute and late toxicity, quality of life and symptoms relief. Although a systematic literature review reported 84.9% (_95%_CI, 75.8–91.5%) overall pain response in LAPC patients treated with SBRT [31], studies comparing the pain-relieving effect of SBRT with other treatment options in this setting are still missing. 

Furthermore, our analysis only included patients undergoing SBRT in a few fractions, or CRT delivered with standard fractionation, and therefore we were unable to compare these treatments with different regimens (e.g., radiotherapy protocols based on moderate hypofractionation). Moreover, our analysis included several and numerically limited subgroups of patients undergoing different chemotherapy regimens (Supplementary Paragraph S2), and this precluded the possibility of comparing results based on systemic therapy protocols. Finally, the exclusion from the analysis of patients who underwent a pancreatectomy before or after treatment did not allow comparisons between the different treatments in terms of resectability.

### 4.3. Future Studies

Future randomized trials should investigate modern CHT regimens as gemcitabine + nab paclitaxel or FOLFIRINOX in combination with SBRT. In particular, these studies should define the LAPC population who might benefit from such aggressive CHT regimens, in combination with modern radiotherapy techniques in the neoadjuvant setting. 

Results on immunotherapy (IT) failed to demonstrate an advantage in terms of outcomes in pancreatic cancer. IT resistance of pancreatic cancer could be due to the biological phenotype of this disease, defined as “cold”, as a result of its non-T cell-inflammation capability. According to the immune modulation effect of radiation therapy, immunoresistance could be overcome through a combination of IT with radiotherapy. Consequently, future studies should investigate the association of IT and radiotherapy as a novel strategy to convert LAPC from a “cold” to a “hot” tumor amenable to IT [32].

Moreover, some epidemiological risk factors [33] such as tobacco smoking, imbalanced diet and various metabolic pathways predisposing the patient to obesity act as negative predictive factors for different outcomes. Future studies are needed to select patients who are more responsive to novel therapies based on several clinical characteristics.

## 5. Conclusions

Even considering these limitations, our analysis suggests that: (i) in daily clinical practice, at least in selected patients, the addition of radiotherapy to CHT is an option to be considered; (ii) in patients suitable for radiotherapy, CRT can be replaced by SBRT, also considering the shorter treatment duration (iii) and the low risk of acute toxicity [12,33,34,35,36,37,38,39].

Future studies in this field should aim to: (i) define predictive models potentially able to define the optimal individualized treatment for patients with LAPC among the available therapeutic options; (ii) compare the therapeutic options in terms of impact on the quality of life, in particular on pain, considering the poor prognosis of these patients, and therefore the palliative aim of treatment; (iii) compare the available therapies in terms of resectability rates after treatment, considering that surgical resection still represents the only possibility of cure for these patients.

## Figures and Tables

**Table 1:** Patients and treatments characteristics.

Variable	Value	Total (%)	CRT (%)	CHT (%)	SBRT (%)	*p*
Age (years)	Median (range)	66 (34–90)	67 (34–90)	63 (44–88)	68 (36–89)	
	≤65	199 (47.5)	140 (47.0)	35 (53.8)	24 (42.9)	0.457
	>65	220 (52.5)	158 (53.0)	30 (46.2)	32 (57.1)	
Gender	M	226 (53.9)	164 (55.0)	31 (47.7)	31 (55.4)	0.546
	F	193 (46.1)	134 (45.0)	34 (52.3)	25 (44.6)	
ECOG	0	167 (39.9)	121 (40.7)	19 (29.2)	27 (48.2)	0.005
	1	131 (31.2)	71 (23.8)	37 (56.9)	23 (41.1)	
	2	33 (7.9)	21 (7.0)	7 (10.8)	5 (8.9)	
	Missing	88 (21.0)	85 (28.5)	2 (3.1)	1 (1.8)	
Tumor site	Head	283 (67.5)	207 (69.5)	43 (66.2)	33 (58.9)	0.004
	Body	105 (25.1)	75 (25.2)	11 (16.9)	19 (33.9)	
	Tail	26 (6.2)	12 (4.0)	10 (15.4)	4 (7.2)	
	Missing	5 (1.2)	4 (1.3)	1 (1.5)	0 (0.0)	
Tumor diameter (cm)	Median (range)	3.9 (1.2–10.0)	3.6 (1.4–10.0)	4.0 (2.0–7.0)	3.9 (1.2–8.7)	
	<3.0	59 (14.1)	44 (14.8)	8 (12.2)	7 (12.5)	0.271
	≥3.0 and <3.9	112 (26.7)	84 (28.2)	9 (13.9)	19 (33.9)	
	≥3.9	248 (59.2)	170 (57.0)	48 (73.9)	30 (53.6)	
Clinical T stage	3	144 (34.4)	117 (39.3)	10 (15.4)	17 (30.4)	0.012
	4	275 (65.6)	181 (60.7)	55 (84.6)	39 (69.6)	
Clinical N stage	0	165 (39.4)	131 (44.0)	0 (0.0)	34 (60.7)	<0.001
	1–2	232 (55.4)	156 (52.3)	54 (98.2)	22 (39.3)	
	Missing	22 (5.2)	11 (3.7)	11 (1.8)	0 (0.0)	
CHT	Gemcitabine-based	238 (56.8)	165 (55.4)	50 (76.9)	23 (41.1)	0.098
	Fluopyrimidine-based	148 (35.3)	123 (41.2)	7 (10.8)	18 (32.1)	
	Others	18 (4.3)	10 (3.4)	8 (12.3)	/	/
	No	15 (3.6)	/	/	15 (26.8)	/
Total dose (Gy)	Median (range)	50.4 (10.8–66.0)	50.4 (10.8–66.0)	/	30.0 (18.0–45.0)	/
Ca 19-9 (U/mL)	Median (range)	235 (1–25,663)	220 (1–25,663)	290 (1–6206)	203 (1–20,000)	0.389
BED_α/β 10 Gy_	Median (range)	59.4 (12.7–115.1)	59.4 (12.7–115.1)	/	48.0 (28.0–78.7)	/
BED_α/β 10 Gy_	<59.4 Gy	109 (26.0)	58 (19.5)	/	51 (91.1)	<0.001
	≥59.4 Gy	245 (58.5)	240 (80.5)	/	5 (8.9)	
	Missing	65 (15.5)	/	65 (100.0)	/	/
Treatment	CRT	298 (71.1)	/	/	/	/
	CHT	65 (15.5)	/	/	/	
	SBRT	56 (13.4)	/	/	/	

BED: Biologically Effective Dose; CHT: chemotherapy; CRT: chemoradiation; ECOG: Eastern Cooperative Oncology Group; SBRT: stereotactic body radiotherapy.

**Table 2:** Univariate and multivariate analysis on LC.

Univariate Analysis						LC Multivariate Analysis		
Variable	Value	1-Year LC (%)	2-Year LC (%)	Median LC (Months)	*p*	HR	95% CI	*p*
Age	≤65	68.3	46.2	21	0.124			
	>65	60.5	39.3	17				
Gender	M	64.3	42.8	18	0.629			
	F	64.0	41.8	20				
ECOG	0	63.8	47.7	21	0.108			
	1	52.6	34.2	14				
	2	62.2	0.0	15				
Tumor site	Head	61.3	37.5	17	0.020			
	Body	71.1	33.5	28				
	Tail	58.8	29.8	17				
Tumor diameter (cm)	<3.0	60.0	39.9	15	0.191			
	≥3.0 and <3.9	57.2	33.1	15				
	≥3.9	68.3	47.3	22				
cT stage	3	63.1	39.9	17	0.695			
	4	64.7	43.7	20				
cN stage	0	69.4	48.9	21	0.081			
	1-2	60.7	39.3	17				
Ca 19-9 (U/mL)	≤235	39.6	23.7	16	0.801			
	>235	42.1	21.5	19				
Treatment	CRT	66.6	45.4	19	<0.001			
	CHT	42.9	13.8	9				
	SBRT	79.0	60.6	NR				
CHT vs. CRT	CRT					0.61	0.37–1.00	0.053
	tumor site: body					0.58	0.35–0.95	0.032
CRT vs. CHT vs. SBRT	CRT					0.56	0.34–0.92	0.022
	SBRT					0.27	0.13–0.54	<0.001
CRT vs. SBRT	tumor site: body					0.53	0.32–0.88	0.015
	SBRT					0.46	0.25–0.83	0.011
CHT vs. CRT + SBRT	tumor site: body					0.54	0.33–0.90	0.019
CHT vs. SBRTT	tumor site: body					0.30	0.11–0.79	0.015

CHT: chemotherapy; CRT: chemoradiation; DMFS: distant metastases-free survival; ECOG: Eastern Cooperative Oncology Group; LC: local control; OS: overall survival; SBRT: stereotactic body radiotherapy.

**Table 3:** Univariate and multivariate analysis on DMFS.

Univariate Analysis						DMFS Multivariate Analysis		
Variable	Value	1-Year DMFS (%)	2-Year DMFS (%)	Median DMFS (Months)	*p*	HR	95% CI	*p*
Age	≤65	56.3	37.8	15	0.539			
	>65	50.9	32.2	13				
Gender	M	57.1	39.4	16	0.061			
	F	49.6	30.1	12				
ECOG	0	55.1	34.2	14	0.824			
	1	53.7	31.3	14				
	2	48.9	29.3	12				
Tumor site	Head	47.8	32.6	12	0.289			
	Body	60.7	35.4	14				
	Tail	73.6	43.6	24				
Tumor diameter (cm)	<3.0	53.9	26.6	13	0.273			
	≥3.0 and <3.9	48.5	27.8	12				
	≥3.9	55.8	40.2	15				
cT stage	3	39.0	25.1	10	<0.001			
	4	61.0	40.0	16				
cN stage	0	53.5	40.9	15	0.274			
	1–2	52.6	33.0	13				
Ca 19-9 (U/mL)	≤235	33.5	26.8	13	0.930			
	>235	31.9	22.8	13				
BED (α/β 10 Gy)	<59.4 Gy	47.6	20.1	12	0.039			
	≥59.4 Gy	55.0	41.4	16				
Treatment	CRT	52.2	34.8	13	0.819			
	CHT	59.1	42.2	15				
	SBRT	55.6	24.0	14				
CRT vs. CHT vs. SBRT	cT4					0.68	0.47–1.00	0.056

**Table 4:** Univariate and multivariate analysis on OS.

Univariate Analysis						OS Multivariate Analysis		
Variable	Value	1-Year OS (%)	2-Year OS (%)	Median OS (Months)	*p*	HR	95% CI	*p*
Age	≤65	61.4	31.0	16	0.177			
	>65	60.0	22.2	14				
Gender	M	61.1	25.9	15	0.605			
	F	60.2	26.6	15				
ECOG	0	72.8	39.1	19	0.007			
	1	58.2	23.9	15				
	2	56.1	10.9	13				
Tumor site	Head	56.9	21.1	14	0.025			
	Body	65.8	32.6	17				
	Tail	76.2	54.3	28				
Tumor diameter (cm)	<3.0	64.2	30.5	14	0.088			
	≥3.0 and <3.9	70.1	33.0	16				
	≥3.9	62.0	22.3	15				
cT stage	3	52.7	19.7	13	0.078			
	4	65.0	29.9	16				
cN stage	0	68.4	23.9	16	0.068			
	1-2	55.8	26.6	14				
Ca 19-9 (U/mL)	≤235	35.5	12.2	18	0.042			
	>235	25.5	4.7	15				
Treatment	CRT	62.2	29.1	15	<0.001			
	CHT	36.0	12.2	10				
	SBRT	81.9	27.1	19				
CHT vs. CRT	CRT					0.44	0.27–0.70	<0.001
CRT vs. CHT vs. SBRT	CRT					0.44	0.28–0.70	<0.001
	SBRT					0.40	0.22–0.74	0.003
CRT vs. SBRT	tumor site: tail					0.30	0.11–0.82	0.019
CHT vs. CRT + SBRT	tumor site: tail					0.30	0.11–0.82	0.019

## Data Availability

The data presented in this study are available on request from the corresponding author.

## Supplementary Materials

The following supporting information can be downloaded at: https://www.mdpi.com/article/10.3390/curroncol30060427/s1, Figure S1: Flow chart of inclusion and exclusion procession; Paragraph S2: Detailed description of chemotherapy protocols used in the three analyzed cohorts.

## List of Abbreviations

BED: biologically effective dose; CI: confidence interval; CHT: chemotherapy; CRT: chemoradiation; DMFS: distant metastasis free survival; ECOG: Eastern Cooperative Oncology Group; HR: Hazard ratio; IT: immunotherapy; LAPC: locally advanced pancreatic cancer; LC: local control; OS: overall survival; SBRT: stereotactic body radiotherapy.
