# Supplementary material for: Comparative Effectiveness of Chemotherapy Alone Versus Radiotherapy-Based Regimens in Locally Advanced Pancreatic Cancer: A Real-World Multicenter Analysis (PAULA-1)
Source: Curr Oncol. 2023 Jun 10;30(6):5690–703. doi: 10.3390/curroncol30060427 (PMC10296903; doi:10.3390/curroncol30060427)
Supplement: Supplementary file 1 [file curroncol-30-00427-s001.zip › Paragraph_S2.pdf]

SUPPLEMENTARY MATERIAL

Paragraph S2: Detailed description of chemotherapy protocols used in the three analyzed cohorts

CHT cohort: 65 patients

- 51 (78.5%), gemcitabine,
- 7 (10.7%), FOLFIRINOX,
- 2 (3.1%), FOLFOX,
- 2 (3.1%), GEMOX,
- 2 (3.1%), Gemcitabine + Nab-Paclitaxel,
- 1 (1.5%), Oxaliplatin.

Stereotactic Body Radiotherapy cohort: 56 patients

- Chemotherapy before SBRT to 18 (32.1%) patients
- Chemotherapy after SBRT to 10 (17.9%) patients
- Chemotherapy before and after SBRT to 13 (23.2%) patients.
- SBRT without chemotherapy to 15 (26.8%) patients

Chemotherapy regimens were based on

- Gemcitabine (43.5%),
- Gemcitabine plus Nab-Paclitaxel (38.7%),
- FOLFIRINOX (17.8%).

Concurrent chemoradiation cohort: 298 patients

<u>Chemotherapy before chemoradiation to 177 (59.4%) patients</u>

- 95 (53.7%), Gemcitabine,
- 55 (31.1%), GEMOX
- 10 (5.5%), FOLFIRONOX
- 8 (4.5%), Gemcitabine + Cisplatin
- 4 (2.3%), Gemcitabine + Capecitabine
- 4 (2.3%), FOLFOX
- 1 (0.6%), Gemcitabine + Nab-Paclitaxe

<u>Concurrent chemotherapy to 298 (100.0%) patients</u>

- 137 (46.0%), Gemcitabine
- 92 (30.9%), Capecitabine
- 31 (10.4%), FOLFIRINOX
- 28 (9.4%), GEMOX
- 6 (2.0%), Gemcitabine + Cisplatin
- 2 (0.7%), Gemcitabine + Capecitabine
- 1 (0.3%), Tegafur
- 1 (0.3%), Oxaliplatin

Chemotherapy after concurrent chemoradiation to 37 (12.4%) patients

- 27 (73.0%), Gemcitabine
- 4 (10.8%), GEMOX
- 3 (8.1%), FOLFOX
- 1 (2.7%), Capecitabine
- 1 (2.7%), FOLFIRINOX
- 1 (2.7%), Gemcitabine + Capecitabine

Treatment completion details:

-Chemotherapy alone: 52/65 (80.0%)

-Chemoradiation: 256/290 (88.3%)

-Stereotactic radiotherapy: 54/56 (96.4%)
