# Supplementary figures and images for: Comparative Effectiveness of Chemotherapy Alone Versus Radiotherapy-Based Regimens in Locally Advanced Pancreatic Cancer: A Real-World Multicenter Analysis (PAULA-1)
Source: Curr Oncol. 2023 Jun 10;30(6):5690–703. doi: 10.3390/curroncol30060427 (PMC10296903; doi:10.3390/curroncol30060427)

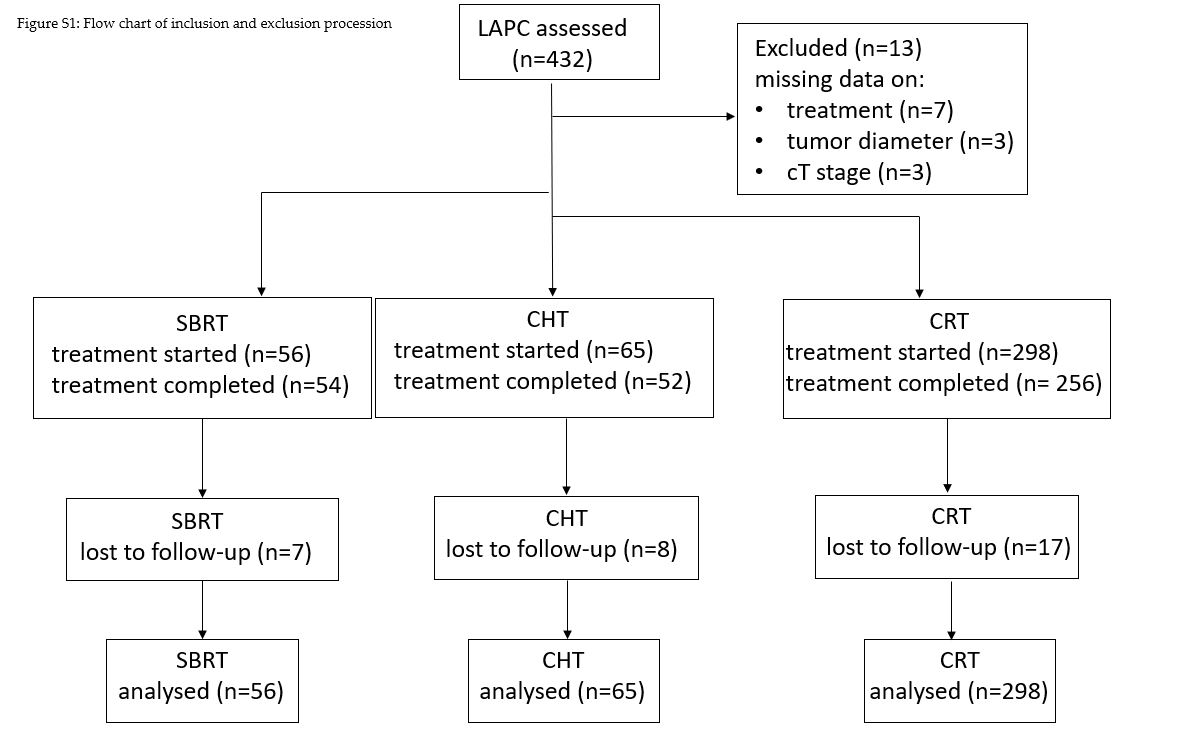

Supplement: Supplementary file 1 [file curroncol-30-00427-s001.zip › Figure_S1.JPG]
